# Mistic: An open-source multiplexed image t-SNE viewer

**DOI:** 10.1016/j.patter.2022.100523

**Published:** 2022-06-02

**Authors:** Sandhya Prabhakaran, Chandler Gatenbee, Mark Robertson-Tessi, Jeffrey West, Amer A. Beg, Jhanelle Gray, Scott Antonia, Robert A. Gatenby, Alexander R.A. Anderson

**Affiliations:** 1 Department of Integrated Mathematical Oncology, H. Lee Moffitt Cancer Center and Research Institute, Tampa, FL 33612, USA; 2 Departments of Immunology and Thoracic Oncology, H. Lee Moffitt Cancer Center and Research Institute, Tampa, FL 33612, USA; 3 Department of Radiation Oncology, H. Lee Moffitt Cancer Center and Research Institute, Tampa, FL 33612, USA; 4 Department of Medicine, Duke University School of Medicine, Durham, NC 27710, USA

**Keywords:** multiplexed images, visualization, t-SNE viewer, UMAP viewer, Mistic, cancer, NSCLC, lung cancer, endometrial cancer, t-CyCIF, CyCIF, CODEX, PerkinElmer Vectra, data exploration, immune landscape, immunotherapy, data analysis

## Abstract

Understanding the complex ecology of a tumor tissue and the spatiotemporal relationships between its cellular and microenvironment components is becoming a key component of translational research, especially in immuno-oncology. The generation and analysis of multiplexed images from patient samples is of paramount importance to facilitate this understanding. Here, we present Mistic, an open-source multiplexed image t-SNE viewer that enables the simultaneous viewing of multiple 2D images rendered using multiple layout options to provide an overall visual preview of the entire dataset. In particular, the positions of the images can be t-SNE or UMAP coordinates. This grouped view of all images allows an exploratory understanding of the specific expression pattern of a given biomarker or collection of biomarkers across all images, helps to identify images expressing a particular phenotype, and can help select images for subsequent downstream analysis. Currently, there is no freely available tool to generate such image t-SNEs.

## Highlights

• Mistic is a multiplexed image t-SNE viewer
• Mistic enables the simultaneous viewing of multiple 2D images
• This grouped overview allows visualization of existing patterns in the data
• Mistic supports images from Vectra, CyCIF, t-CyCIF, and CODEX

## The bigger picture

A crucial component of translational research is in exploiting tumor tissue for diagnostic or prognostic purposes. We believe this can best be achieved through a deeper understanding of the complex ecology of a tumor tissue and the spatiotemporal relationships between its cellular and microenvironment components. Multiplexed images from patient samples facilitate this understanding. We present Mistic, an open-source multiplexed image t-SNE viewer that enables the simultaneous viewing of multiple 2D multiplexed images to provide an overall visual preview of the entire dataset. This allows an exploratory understanding of underlying patterns in the data such as the specific expression pattern of a given biomarker across all images. Currently, there is no free tool to generate such image t-SNEs. Mistic aims to fill this gap by providing an easy to implement tool with simple functionality to view multiple images at once. Mistic supports images from Vectra, CyCIF, t-CyCIF, and CODEX.

## Teaser

Multiplex imaging of tissues allows the simultaneous imaging of multiple biomarkers on a tissue specimen of interest and is a critical tool for clinical cancer diagnosis and prognosis. A common way to visualize and better understand such multiplexed images is to utilize dimensionality reduction (DR) methods, where each image is abstracted as a point in the reduced space. We developed Mistic to enable the simultaneous viewing of multiple 2D multiplexed images by combining DR, image processing, and GUI programming.

## Introduction

Multiplex imaging of tissues, which allows the simultaneous imaging of multiple biomarkers on a tissue specimen of interest, is a critical tool for clinical cancer diagnosis and prognosis. Historically, patient tissue samples stained with hematoxylin and eosin have been used as the gold standard for tumor diagnosis by indicating the presence of tumors and their grade.^1^, ^2^, ^3^ With the advent of immunohistochemical (IHC)^4^ staining and the flourishing of multiplexed imaging approaches that leverage IHC, immunofluorescence (IF), fluorescence *in situ* hybridization (FISH),^5^^,^^6^ multiplexed ion beam imaging (MIBI),^7^ cyclic labeling such as co-detection by indexing (CODEX),^8^ cyclic immunofluorescence (CyCIF),^9^ and imaging mass cytometry (IMC),^10^ there is a wealth of potential data to be gleaned from a single section of tissue. Biomarkers can be observed and quantified with their tissue context completely conserved. Due to the multidimensional nature of the data from these multiplexed images, analysis requires computational pipelines to both interrogate and study how the tissue architecture, spatial distribution of multiple cell phenotypes, and co-expression of signaling and cell cycle markers are related and what patterns might exist.

There are several commercial software platforms available for quantifying and analyzing multiplex image data, for example, Imaris (from Oxford Instruments),^11^ Amira (from Thermo Fisher Scientific),^12^ and Halo (from Indica Labs).^13^^,^^14^ There are also open-source software platforms, for instance, ImageJ,^15^ CellProfiler,^16^ V3D,^17^ BioImageXD,^18^ Icy,^19^ FIJI,^20^ and QuPath^21^ for the analysis of two dimensional (2D) biological images. Most of these platforms allow for a single 2D image to be examined at any one time.

A common way to visualize and better understand multidimensional data, such as that coming from multiplex images, is to utilize dimensionality reduction methods such as uni-form manifold approximation and projection (UMAP)^22^ or t-distributed stochastic neighbor embedding (t-SNE),^23^ where each image is abstracted as a dot in the reduced space. These approaches are especially useful when combined with clustering methods (e.g., Gaussian mixture models [GMM],^24^^,^^25^ Louvain,^26^ and Leiden^27^) that can highlight key aspects of the data. While utilizing these approaches in our own work dealing with multiplexed images of non-small cell lung cancer (NSCLC) tumors, we realized that there could be a significant benefit to visualizing the actual tissue samples behind a UMAP or t-SNE scatter projection, thus giving rise to an “image t-SNE.” In our specific application, inspection of the images that constituted each spatially segregated cluster revealed cluster-specific biomarker patterns that, along with the tumor phenotypes, could be mapped succinctly to the therapy response of each patient. Thus, the image t-SNE rendering aided both our understanding and intuition that there exist distinct tumor patterns that guide the clustering, and that these patterns can potentially inform why a specific therapeutic response emerged, leading to further biological insights.

Motivated by the usefulness of the image t-SNE in our work and in our recent analysis of endometrial cancer,^28^ which we discuss in section tissue microarray cores for endometrial cancer, we have developed Mistic, an open-source multiplexed image t-SNE viewer that enables the simultaneous viewing of multiple 2D images rendered using multiple layout options to provide an overall visual preview of the entire dataset. In particular, the positions of the images can be taken from t-SNE or UMAP coordinates. This grouped view of the images further aids an exploratory understanding of the biomarkers’ specific expression pattern across all images, helping to identify images expressing a particular phenotype or to select images for subsequent downstream analysis. Currently there is no freely available tool to generate such image t-SNEs (see Table 1). Software such as BioImageXD and Icy offer do offer a “gallery” or “stack montage” option, where a multichannel image is split into its individual channels to be viewed at once. Mistic is distinct in that *multiple* multichannel images can be processed and rendered at once using either user-pre-defined coordinates (e.g., from t-SNE or UMAP analysis), random coordinates, or using a grid layout. Mistic is agnostic as to how the t-SNE/UMAP 2D coordinates are generated by the user. Since t-SNE/UMAP rendering of a dataset is closely aligned to the specific research question, Mistic allows the user to utilize either t-SNE or UMAP projections—or newer ones as they emerge—based on the user’s specific question.

**Table 1:** Comparison of Mistic with commercial and open-source imaging software

#	Software	Image type	Multiple image viewer (M)/stack montage (S)^a^	Render images using t-SNE coordinates	Render images randomly	Open source	Image zoom	Image analysis^b^
1	Mistic	2D multiplexed images	both	✓	✓	✓	✓^c^	✗
2	Imaris^11^	3D/4D microscopy images	M	✗	✗	✗	✓	✓
3	Amira^12^	2D-5D CT, MRI, 3D microscopy,	S	✗	✗	✗	✓	✓
4	Halo^13^^,^^14^	2D multiplexed images	S	✗	✗	✗	✓	✓
5	Volocity^29^^,^^30^	3D microscopy images	S	✗	✗	✗	✓	✓
6	ImageJ^15^	2D multiplexed images	S	✗	✗	✓	✓	✓
7	CellProfiler^16^	2D multiplexed images	both	✗	✗	✓	✓	✓
8	V3D^17^^,^^31^	3D microscopy image stacks	S	✗	✗	✓	✓	✓
9	BioImageXD^18^	microscopy images	S	✗	✗	✓	✓	✓
10	Icy^19^	2D-5D CT, MRI, 3D microscopy	S	✗	✗	✓	✓	✓
11	FIJI^20^	2D multiplexed images	S	✗	✗	✓	✓	✓
12	QuPath^21^	2D multiplexed images	S	✗	✗	✓	✓	✓
13	ml4a^32^	2D images	M^f^	✓	✗	✓	✓^c^	✗
14	Mirador^33^	2D images	M^d^^,^^f^	✗	✗	✓	✓	✗
15	OpenSeaDragon (OSD)^34^	2D images	M^f^	✗	✓	✓	✓	✓^e^

Currently available image analysis software that allow single multiplexed images to be viewed and analyzed, compared with Mistic that allows multiple multiplexed images to be viewed simultaneously, either as an image t-SNE, grid view, or using random coordinates. Mistic has the potential to be integrated into these existing image analysis pipelines as a first step to generate an all-image preview.

a Multiple image viewer: if the images are multiplexed, we mean that each image is a multichannel image itself, unlike a single multichannel image being visualized with its individual channels separate, which we refer to as stack montage. If the images are not multiplexed but just single 2D images, then multiple image viewer would mean viewing multiple of these single 2D images. See footnote (f).

b We refer to image filtering functionalities such as, but not limited to, adjusting brightness, luminosity, sharpness, or quantitative image analysis such as detecting and measuring cells (segmentation).

c Zooming in and out is possible over the entire canvas consisting of multiple images, although individual images cannot be zoomed in or out.

d Limited to two images placed side by side.

e Only image filtering (https://pages.nist.gov/OpenSeadragonFiltering/).

f Note that here the multiple images consist of multiple single 2D images (not multiplexed).

In section image t-SNE-based visualization of multiplexed images from a NSCLC cohort shows marker expression clustering across different patient response groups, we illustrate the importance of visualizing multiplexed images using an image t-SNE in the context of NSCLC. In section Mistic: an open-source multiplexed image t-SNE viewer, we describe Mistic and its features, in more detail. We run Mistic on several datasets using different data formats and describe these results in section generalizability and scalability experiments. In section discussion, we compare Mistic to alternative approaches and conclude with future work. Further details of the code and data can be found in section data and code availability.

## Results

### Image t-SNE-based visualization of multiplexed images from an NSCLC cohort shows marker expression clustering across different patient response groups

We computationally analyzed 92 7-stain PerkinElmer Vectra images from nine patients with advanced/metastatic NSCLC with progression.^35^ They were treated with an oral HDAC inhibitor (vorinostat) combined with a PD-1 inhibitor (pembrolizumab). Tumor biopsies were collected from all patients both pre- and on-treatment. Of the nine patients, four qualified as “Response 1” and five as “Response 2,” where responses are based on how the tumors have progressed per the RECIST classification.^36^ There are 34 images from patients having Response 1 and 58 images from patients classified as Response 2. Note that we have labeled the clusters, markers, patients, and responses in a generic fashion, since the biological conclusions arising from these data are not the purpose of this work.

We extracted the cell segments per field of view (FoV), built a count matrix with cells as rows and markers as columns, and clustered the count matrix to identify heterogeneous cell types, in particular tumor and immune cells. From these cell types, we automatically demarcate tumor-rich regions, across images.

**Figure 1:**
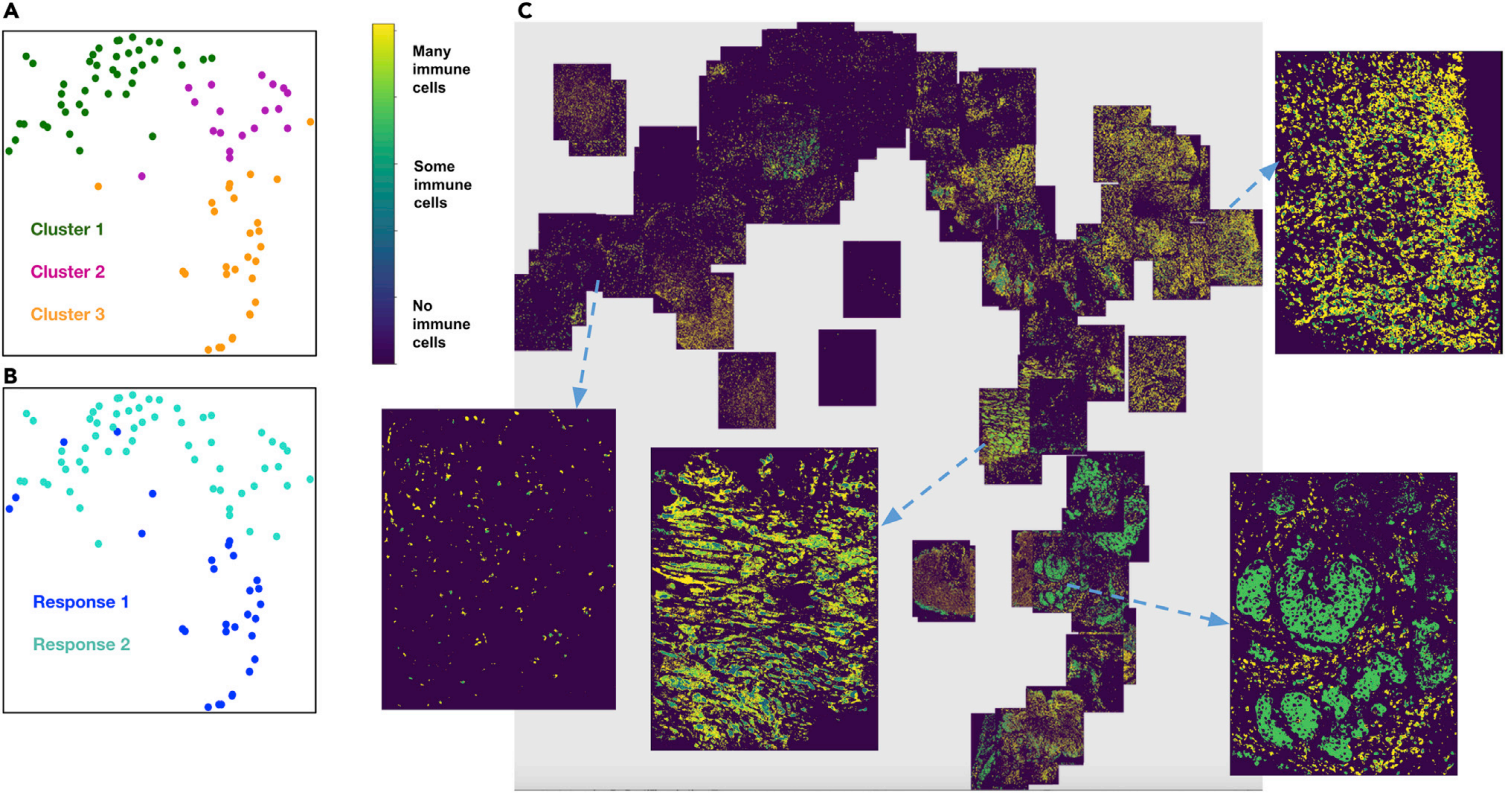
Viewing multiple NSCLC multiplexed images simultaneously Response 1 and Response 2 patients have significantly different cellular compositions. (A) 2D t-SNE plot showing three clusters annotated with the differentially expressed markers per cluster. Clusters are obtained using the tumor-immune cell counts at the tumor border where the borders are estimated using convex hulls approximations. (B) Same t-SNE as in (A) depicting the disease response spread. (C) Image t-SNE using same t-SNE coordinates as in (A) illustrating the gradient of immune cells across images. A higher colocalization of immune cells (shown in green) is seen for Response 1 patients.

To further quantify the tumor-immune cell colocalization at the tumor border, we cluster the tumor-immune cells at the tumor border using a GMM.^24^^,^^25^ The input matrix to the GMM consists of cells (as rows) and their marker distribution as features (columns). The rows of the input matrix are ordered based on the cluster assignments, and the *Z* score of the marker expression (columns) is averaged over vectors per cluster representing a cell type (rows). Those markers that have a higher *Z* score per cluster are identified as the differentially expressed markers for that cluster. The clusters are visualized using a standard 2D t-SNE plot where each point represents an image (Figures 1A and 1B). The differentially expressed markers for each of the three clusters are shown in Figure 1A, and the corresponding patient responses of either Response 1 or Response 2 categories, which are known *a priori*, are depicted in Figure 1B. We see that there is a higher colocalization of different sets of markers for Response 1 and Response 2 patients, respectively (Figures 1A and 1B), indicating underlying structural differences between different patient response groups.

To better understand how these clusters relate to the actual images, we generated an image t-SNE (Figure 1C) where each dot in the t-SNE of Figures 1A and 1B is replaced with its corresponding multiplexed image. This arrangement of images projected as an image t-SNE clearly highlights the difference in immune cell abundance across Response 1 and Response 2 patient groups.

### Mistic: An open-source multiplexed image t-SNE viewer

In order to facilitate the generation and manipulation of image t-SNEs, we developed an image t-SNE viewer called Mistic (multiplexed image t-SNE viewer). Mistic allows the simultaneous viewing of multiple multiplexed images, where images can be arranged using either pre-defined coordinates (e.g., t-SNE or UMAP), randomly generated coordinates, or a grid view. Mistic is written in Python and uses Bokeh,^37^ which is a Python library for creating interactive visualizations for modern web browsers, along with JavaScript. Mistic has the capability to load and display multiple multiplexed images along with the metadata for the images. In Table 2, we provide the different imaging formats and number of images Mistic can be scaled to. It produces publication-ready outputs that can be saved in PNG format. Additionally, it can be used as the initial image viewer for exploratory image analysis before switching to more comprehensive (but single-image) viewers such as ImageJ,^15^ Fiji,^20^ and QuPath.^21^

**Table 2:** Summary of the different datasets Mistic is tested on

Dataset	Format	Dimensions	Size per image	Number of images	Thumbnail size per TIFF	Image generated by
NSCLC FoVs	7-marker TIFF	7 × 1,344 × 1,008	10–50 MB	92	<100 kB	Perkin Elmer Vectra
Lung cancer lymph node^38^, ^39^, ^40^	OME-TIFF	44 × 10,101 × 9,666	13 GB	70	<22 MB	t-CyCIF
Lung cancer primary^38^, ^39^, ^40^	OME-TIFF	44 × 14,447.5 × 11,100.5	322 MB per channel (21.22 GB total file size)	44 slides	<10 MB	t-CyCIF
Endometrial cancer^28^	PNG (generated from 7-marker TIFF)	950 × 1,200	250–1 MB	210	<300 kB	Perkin Elmer Vectra
Human FFPE tonsil^41^	OME_TIFF	32 × 6,720 × 5,040	67.7 MB per channel (2.17 GB total file size)	32 slides	<3.7MB	CODEX
Human FFPE breast adenocarcinoma^41^	OME_TIFF	64 × 5,040 × 9,408	6.07 GB	88	8.2 MB	CODEX
Human FFPE tonsil demo data	QPTIFF	16 × 2,760 × 2,070	2.45 GB	105	1.1 MB	CODEX
Human colorectal carcinoma^42^	OME_TIFF	4 × 1,344 × 1,792	7.4 MB	42	∼600 KB	CyCIF

Overview of different datasets Mistic has been tested on.

**Figure 2:**
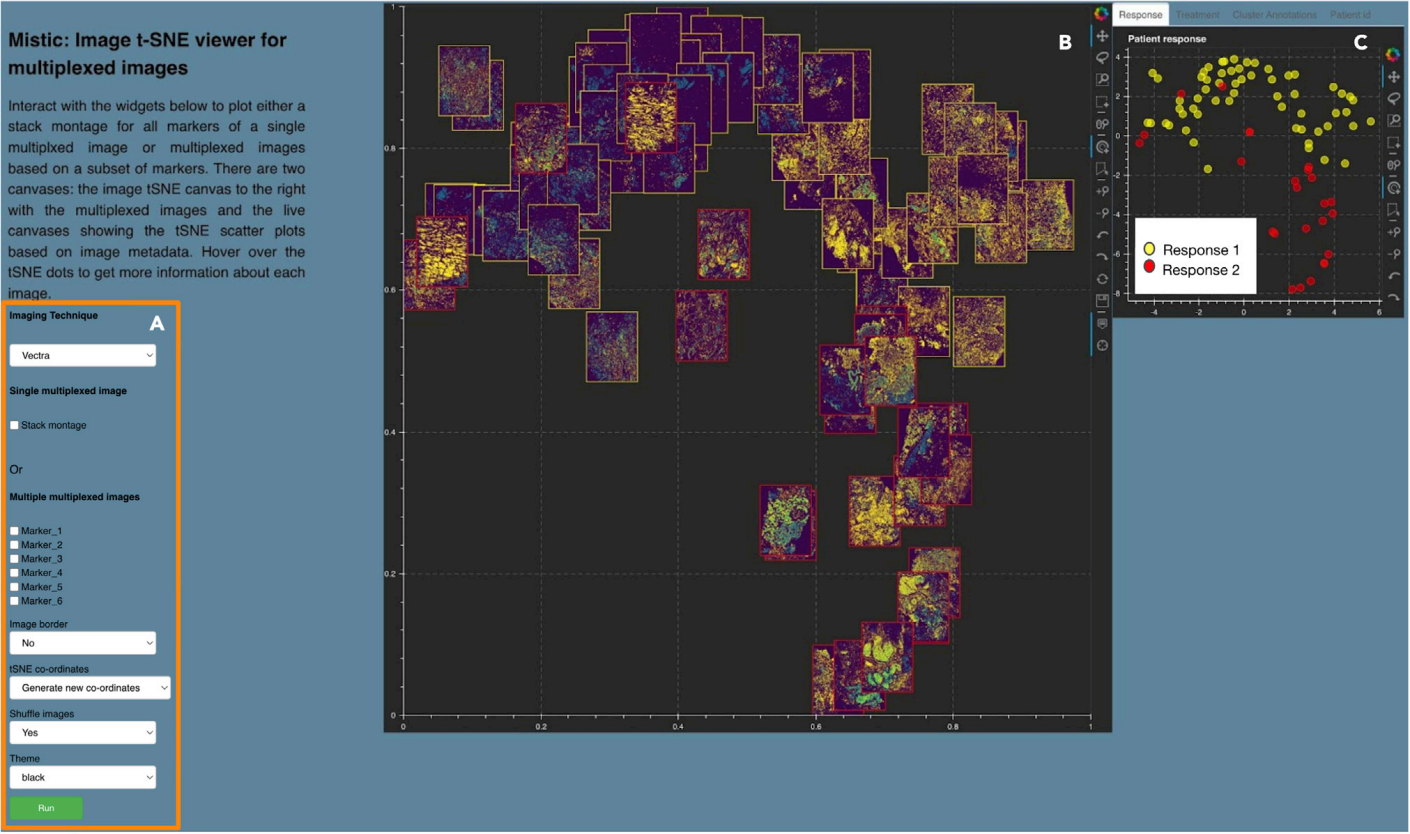
Mistic GUI (A) User-input panel where imaging technique choice, stack montage option, or markers can be selected, images borders can be added, new or pre-defined image display coordinates can be chosen, and a theme for the canvases can be selected. (B) Static canvas showing the image t-SNE colored and arranged per user inputs. (C) Live canvas showing the corresponding t-SNE scatterplot where each image is represented as a dot. The live canvas has tabs for displaying additional information per image. Metadata for each image can be obtained by hovering over each dot.

Mistic provides many of the standard image-viewing features that users have come to rely on and expect, through a user-input panel and two canvases. The user-input panel (Figure 2A) allows the user to select between (1) the stack montage view where all the markers of a single multiplexed image can be viewed simultaneously or (2) the multiple image view. For the latter, user can choose markers for rendering the multiplexed images, optional image borders, the arrangement of the images by coordinates or grid, and the option to shuffle the order of image rendering for overlapping images. An overall color theme for Mistic can be chosen from black, blue, and gray. The user can also choose the imaging technique used to generate the images such as Vectra, CyCIF, t-CyCIF, or CODEX (PhenoCycler). Mistic further provides two canvases for image t-SNE rendering: a static canvas showing the image t-SNE with all the multiplexed images (Figure 2B), which is generated based on user preferences, and a live canvas depicting the corresponding t-SNE scatterplot that uses the metadata from the images, where each image is represented as a dot (Figure 2C). We explain the two canvases in detail in the following subsections (image t-SNE rendered through the static canvas and metadata rendered through the live canvas).

#### Image t-SNE rendered through the static canvas

**Figure 3:**
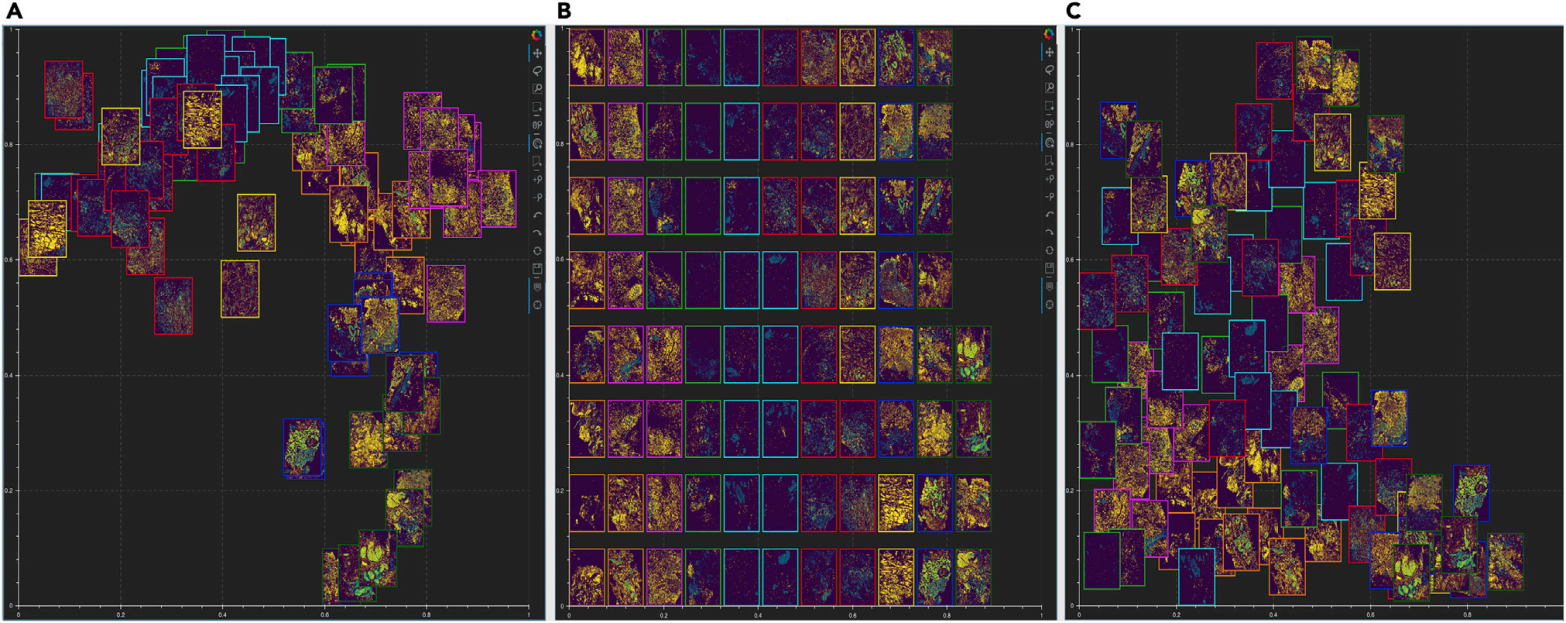
Image layout in Mistic’s static canvas (A–C) Based on user-defined t-SNE coordinates; (B) vertical rows; (C) randomly placed.

**Figure 4:**
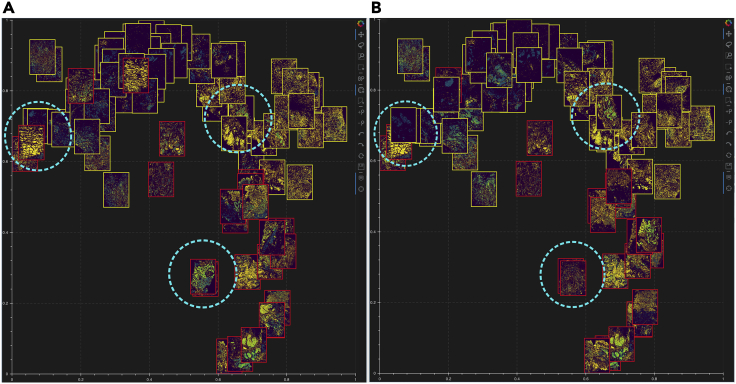
Shuffle option provided by Mistic (A and B) Mistic can shuffle the order in which images are rendered on the static canvas. Examples of images being shuffled between two renderings (A) and (B) are marked in dotted circles.

To view the multiplexed images simultaneously, Mistic offers the user the ability to choose from three different image layouts (see Figure 3): (1) t-SNE layout based on user-pre-defined coordinates; (2) vertical grid arrangement of all images; (3) random layout based on coordinates that Mistic generates. Depending on the specific layout chosen, the live canvas will be updated accordingly (see next section metadata rendered through the live canvas). The user can also opt to shuffle the front-to-back order in which images are rendered, as shown in Figure 4; this is particularly useful when there are many overlapping images. These options can be chosen from the user-input panel (Figure 2A).

Additional options available to users include (1) choosing the canvas color theme (black, gray, or dark blue) and (2) applying borders around images to highlight specific metadata about the images. In Figure 2B, for the example from our NSCLC cohort, the borders indicate images belonging to either Response 1 (yellow border) or Response 2 (red border) patients. In Figure S1, we show image borders colored based on other metadata provided by the user such as treatment, cluster annotations, or patient IDs.

The user has access to the overall image being rendered in the static canvas at *code/image_tSNE_GUI/static* as .png files.

#### Metadata rendered through the live canvas

**Figure 5:**
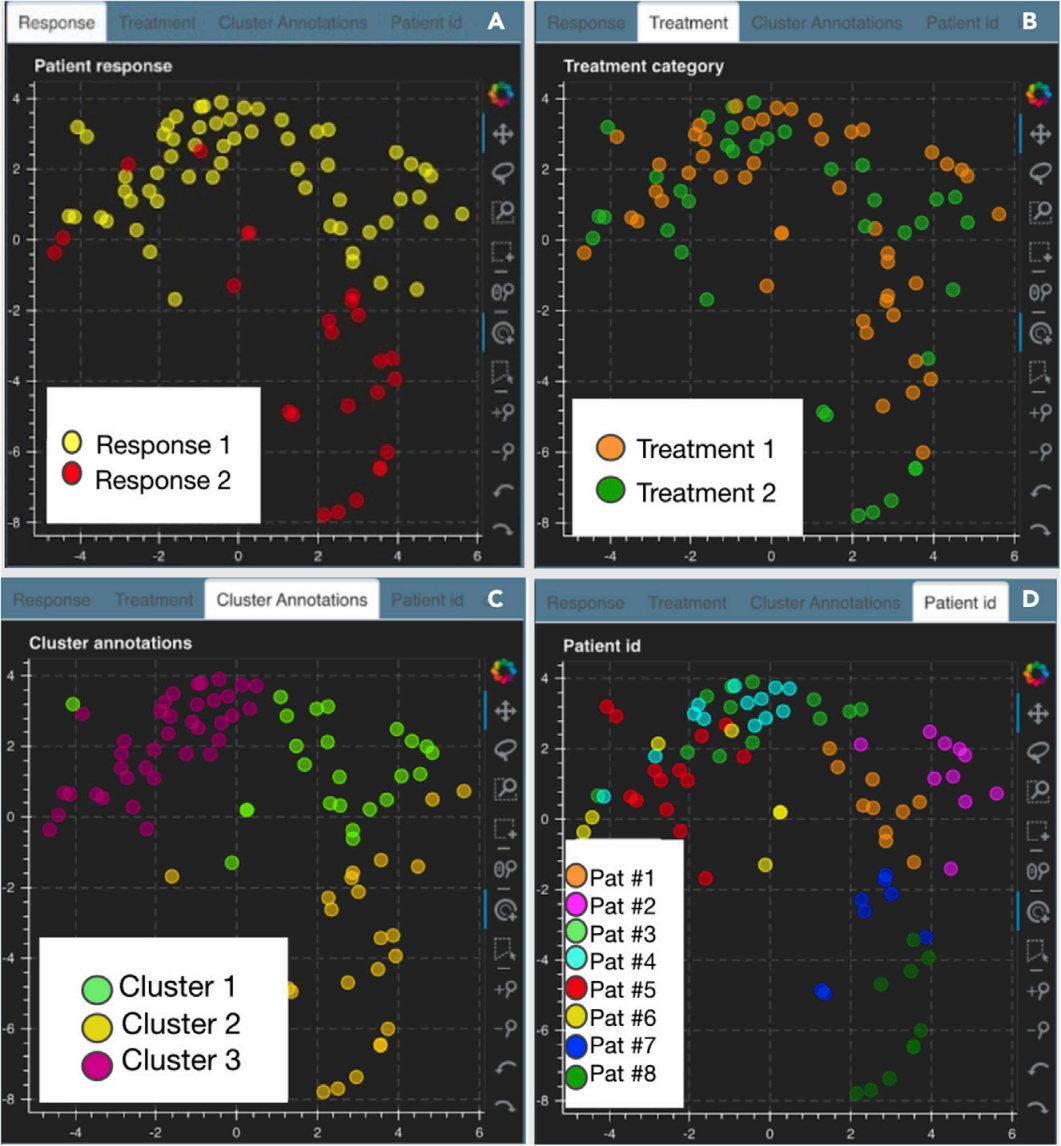
Live canvas of Mistic rendering the t-SNE scatterplot with different image metadata for our NSCLC example (A–D) The metadata consists of response category (A), treatment phase (B), cluster annotations based on marker expression (C), and patient IDs (D).

The live canvas of Mistic offers different metadata renderings of the multiplexed images through t-SNE scatterplots where every multiplexed image is a dot on the scatterplot (Figure 5). In our NSCLC example, Mistic renders the t-SNE scatterplot based on one of the following:

(1) response category of the patients (e.g., based on RECIST classification)
(2) treatment phase (such as pre-treatment or during treatment)
(3) cluster annotations that are based on the differential-expression analysis of the markers
(4) patient distribution

This metadata information may be provided by the user, using appropriate folders provided in Mistic’s code repository, available here: https://github.com/MathOnco/Mistic. If no metadata is provided, the t-SNE scatterplot without any color coding will be rendered.

##### Hover tool for image identification

**Figure 6:**
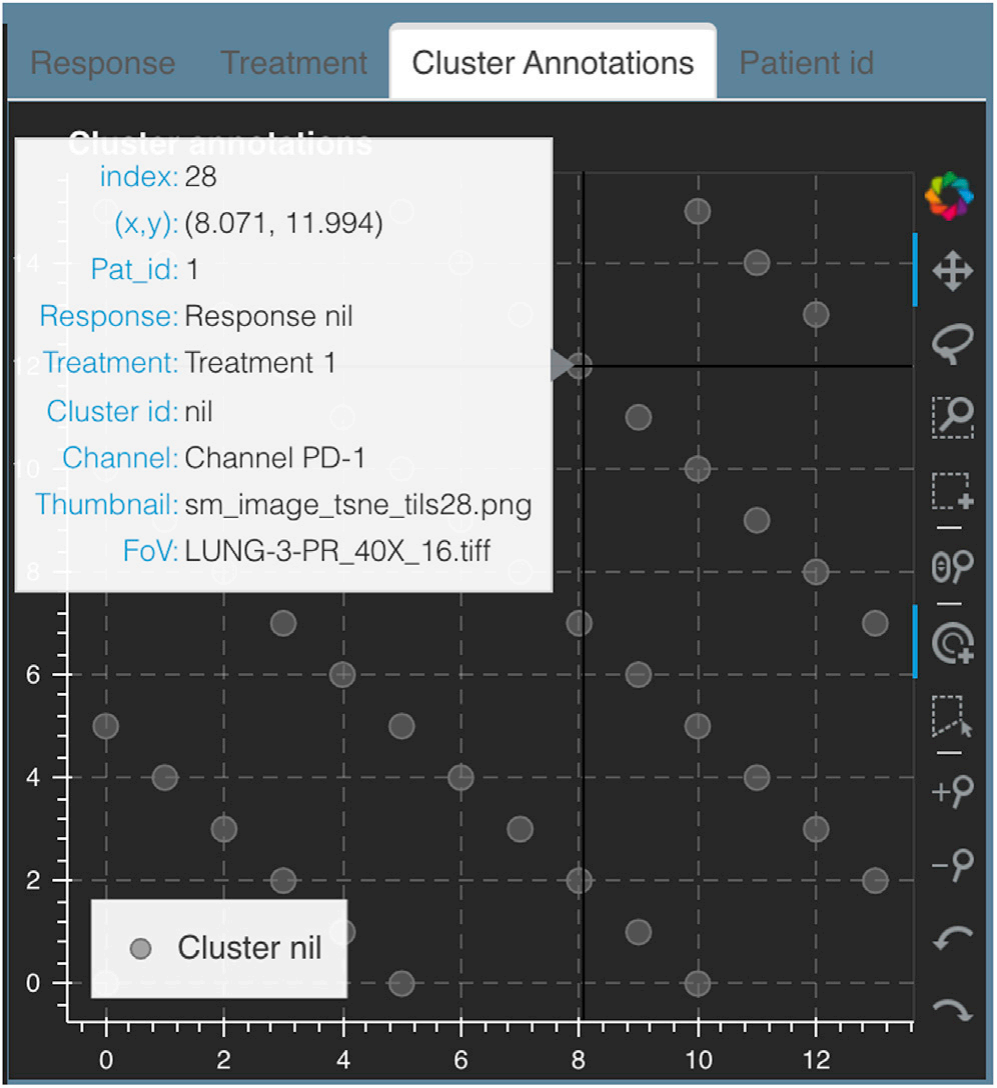
Hover window An example hover window that opens with the hover tool while mousing over a t-SNE dot on any of the live canvases (here shown for “Cluster annotations”). This live canvas is for the stack montage option discussed in section t-CyCIF image of primary lung squamous cell carcinoma.

In order to identify each image in the static canvas, we have a hover functionality built into the live canvases. Hovering over each image provides information such as name of the image, name of the corresponding thumbnail, image coordinates, and all metadata per image (Figure 6).

#### Processing user inputs from Mistic GUI

##### Image processing based on markers selected

Each user-selected marker channel of the multiplexed image is denoised separately. We use the scikit-image^43^ and SciPy^44^ libraries for Python.

1. We use median filtering, which is a nonlinear digital filtering technique, often used to preserve edges while removing noise and improving morphology detection. Function used is *scipy.ndimage.median_filter().*
2. Next, we perform Otsu thresholding, which is an adaptive thresholding for image binarization. This calculates a distribution for the pixel levels on each side of the threshold, i.e., to demarcate pixels that either fall in foreground or background. The aim is to automatically find the threshold value where the sum of foreground and background distribution is at its minimum. Function used is *threshold_otsu()* from scikit-image.
3. Based on the threshold, we close the gaps in the image to refine morphological boundaries. Function used is *closing()* from scikit-image.
4. To sharpen the morphological boundaries, we clear the boundaries using clear_border() from scikit-image.
5. The pixel intensities in each channel are then upweighted to preserve morphology.

The cleaned channels are then combined to form the cleaned multichannel image. The denoised image is stored as an array in the unsigned byte format (“uint8”) to enable easy format conversion.

These are performed in generate_image_tSNE() in main.py in Mistic’s code repository.

##### Inbuilt dimensionality reduction and Bayesian clustering

Mistic will generate both 2D t-SNE coordinates and cluster the images, if the t-SNE coordinates or cluster labels are not provided by the user. Each multiplexed image is abstracted to a vector of length 6 where the entries of the vector are the means of the initial six channels. These vectors are stacked to create a matrix that is input to a t-SNE generation function (sklearn.manifold’s tSNE()) and subsequently clustered using sklearn.mixture’s BayesianGaussianMixture().

##### Border option

An image with a border is created by pasting the cleaned image onto a rectangle with a slightly larger height and width than the cleaned image. The rectangle is filled with a color based on the metadata provided by the user. These are performed in generate_image_tSNE() in main.py.

##### Thumbnail generation

A thumbnail is a concise representation of the original multiplexed image rendered based on user selections. Thumbnails are created for all multiplexed images by downscaling and resizing the height and width of the cleaned image; these are also saved in the *code/output_tiles* folder as .png files. These are performed in generate_image_tSNE() in main.py. There is a provision within the code to generate weighted thumbnails, if required, where weights can be added for each marker channel to differentiate between, for instance, immune markers and tumor markers.

##### Random co-ordinate creation

To generate a set of non-clustered random sample of 2D points, we use a modified version of the “Poisson disc sampling” approach.^45^^,^^46^ These are performed in get_cell_coords(), get_neighbors(), point_valid(), and get_point() in main.py.

Finally, the thumbnails are pasted onto a larger 2D image that gets rendered onto Mistic’s static canvas where the thumbnails are positioned based on pre-defined coordinates (e.g., t-SNE or UMAP), randomly generated coordinates, or as vertical grids.

#### Stack montage

**Figure 7:**
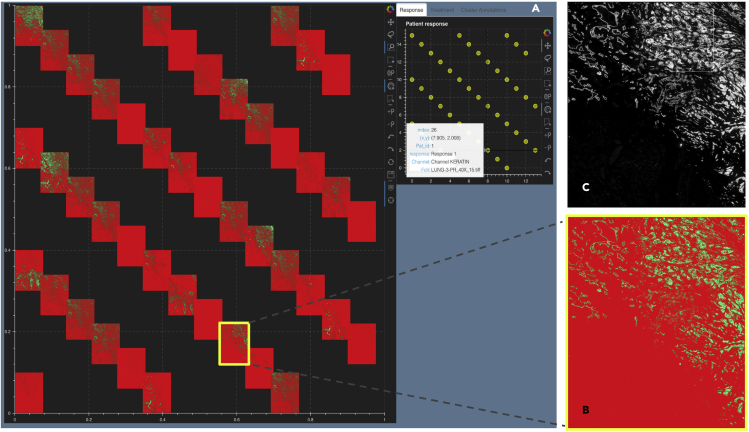
Stack montage from Mistic for the primary lung t-CyCIF data^38^, ^39^, ^40^ with 44 markers (A) The static canvas shows all 44 markers, and the live canvas shows the t-SNE scatterplot. We identify the keratin channel using the live canvas (shown with hover tool details) and highlight the keratin thumbnail in yellow in the static canvas. (B) The zoomed-in keratin thumbnail (file name obtained from the hover tool) and (C) the t-CyCIF image for keratin as viewed using Minerva.^47^ Minerva provides the single marker views for 12 markers, whereas with Mistic, we can view all 44 channels as a montage.

For a single multiplexed image, Mistic provides the user with a stack montage view made up of the individual markers (Figure 7). The user can opt for this to visualize and compare the individual marker channels, explore markers that are visually similar, and detect any potential anomalies when imaging certain marker channels. Note that software such as ImageJ and FIJI provide this option via a plugin along with further image-adjusting functionalities (e.g., brightness, sharpness).

#### Bokeh plot tools

**Figure 8:**
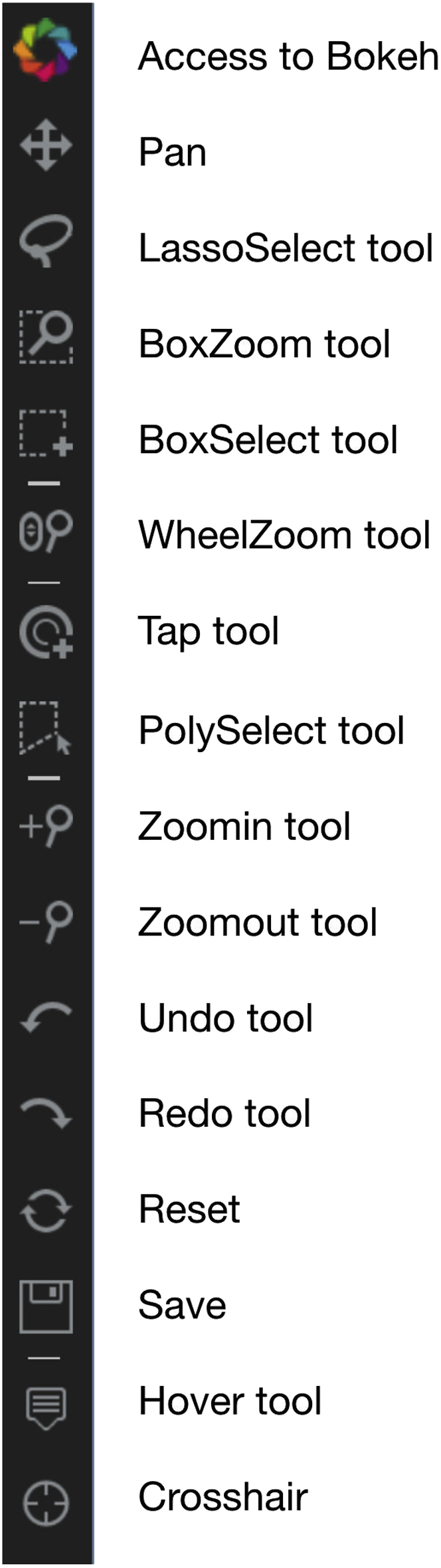
Interactive Bokeh plot tools used in Mistic

Each Mistic canvas uses the interactive Bokeh toolbar to save plots, select regions, and change plot parameters such as zoom level, reset, pan, etc. Figure 8 shows the set of plot tools used. Further documentation of the Bokeh toolbar and how to use it can be found here: https://docs.bokeh.org/en/latest/docs/user_guide/tools.html.

### Generalizability and scalability experiments

#### t-CyCIF image of lung adenocarcinoma metastasis to lymph node

**Figure 9:**
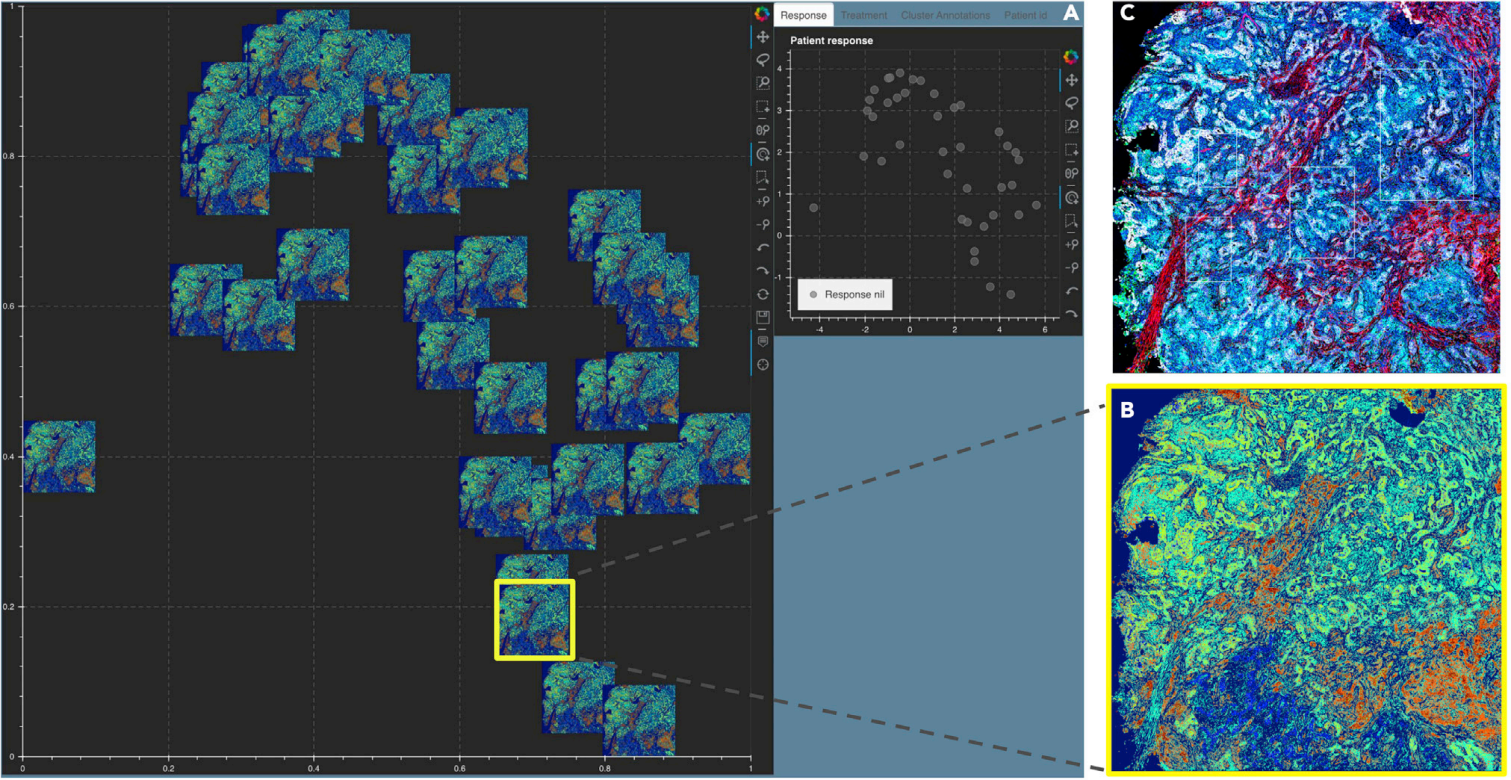
Mistic tested on the 44-channel lung adenocarcinoma lymph t-CyCIF data^38^, ^39^, ^40^ (A) The static canvas shows 40 repeats of the lung t-CyCIF image, arranged in rows. (B) The zoomed-in composite image thumbnail and (C) the t-CyCIF image as viewed using Minerva.^47^ Minerva provides the composite image with five markers (CD45, IBA1, keratin, α-SMA, DNA), whereas Mistic gives the composite image using six markers (CD45, keratin, α-SMA, FoxP3, PD-1, PD-L1).

To show the generalizability of Mistic, we use t-CyCIF data from lung adenocarcinoma metastasized to the lymph node.^38^, ^39^, ^40^ The image is in OME-TIFF format,^48^ 13 GB in size with dimensions 10,101 × 9,666, and it has 44 marker channels. To simultaneously test Mistic for scalability, we created duplicates of this image. Figure 9A shows the Mistic static canvas where 40 duplicate t-CyCIF images with six markers (CD45, keratin, α-SMA, FoxP3, PD-1, PD-L1) are rendered in rows. The zoomed-in composite image thumbnail is shown in Figure 9B with the corresponding composite image as seen in Minerva^47^ for five markers (CD45, IBA1, keratin, α-SMA, DNA) (Figure 9C). Mistic allows the user to choose any number of markers for simultaneous viewing, while Minerva allows up to five markers. In Figures S2–S4, Mistic is shown on 50, 60, and 70 image repeats, respectively, where images are either rendered in rows or randomly.

#### t-CyCIF image of primary lung squamous cell carcinoma

For a single multiplexed image, Mistic provides the user a stack montage view made up of the individual markers. In Figure 7A, we show this option for the t-CyCIF image on primary lung squamous cell carcinoma^38^, ^39^, ^40^ in OME-TIFF format for all 44 marker channels. We highlight the keratin channel in Figure 7B and show the corresponding channel using Minerva^47^ (Figure 7C). Minerva provides single marker views for 12 markers, whereas Mistic renders all 44 channels as a montage.

#### Tissue microarray cores for endometrial cancer

**Figure 10:**
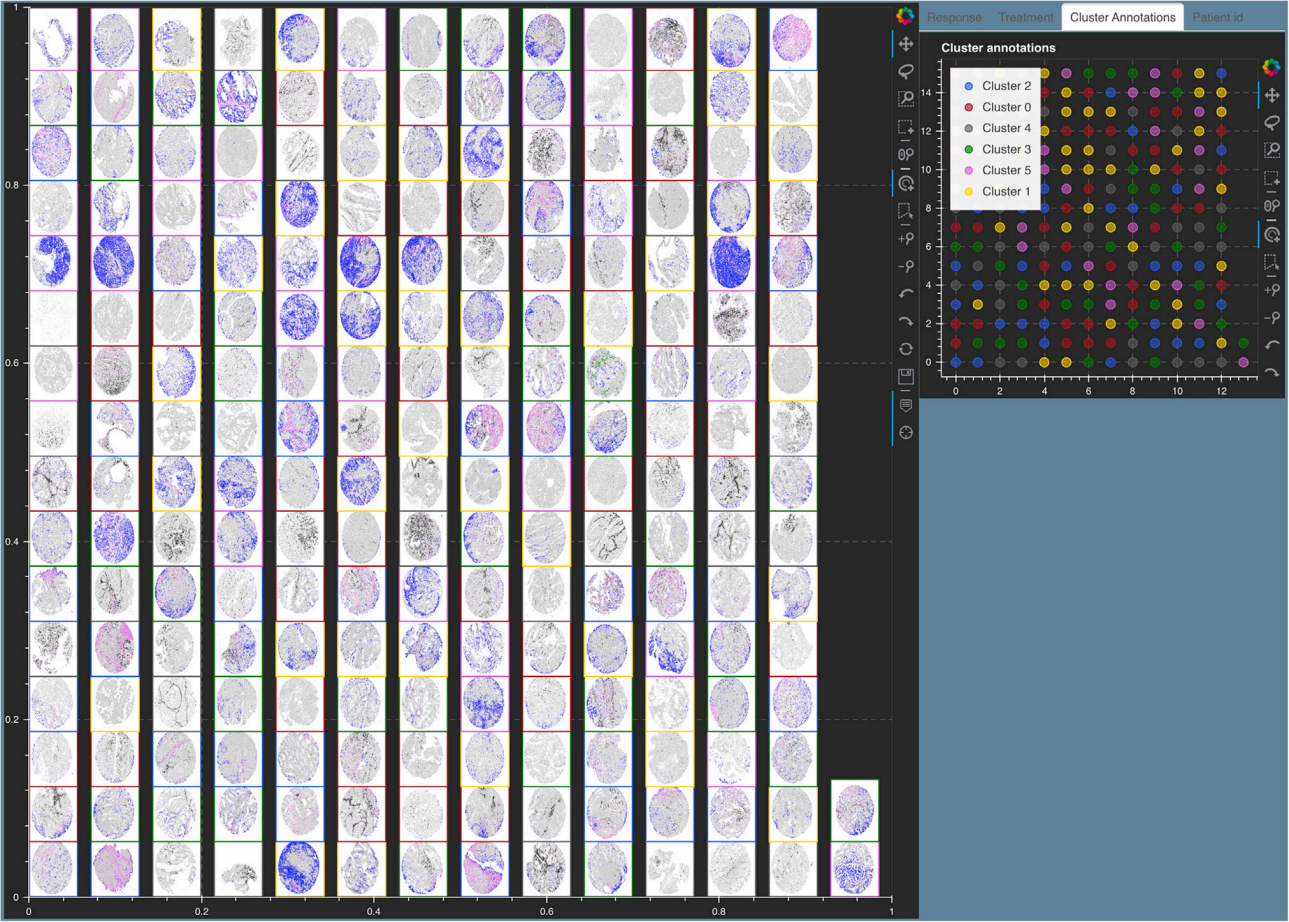
Mistic renders 210 tissue microarray (TMA) cores of endometrial cancer^28^ arranged in rows Each core depicts pIgR on tumor cells (blue), IgA (black), IgG (pink), plasma cells (red), additional B cells (green), and unstained cells (gray). Each core has a border that matches the cluster it belongs to (see the Cluster Annotations live panel).

**Figure 11:**
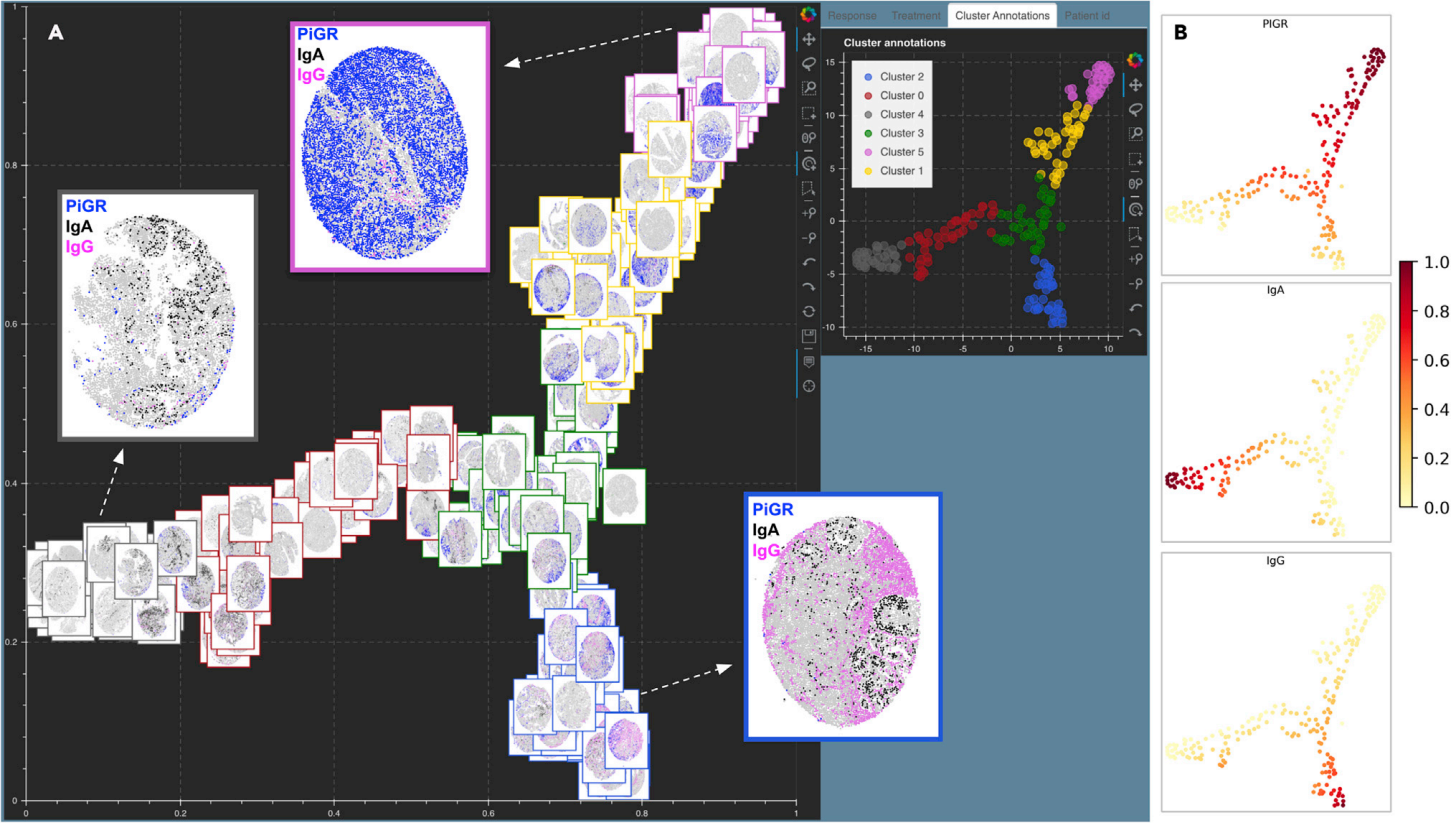
Mistic for 210 TMA cores of endometrial cancer^28^ (A) Image t-SNE rendering using Mistic for 210 TMA cores of endometrial cancer.^41^ Each core depicts pIgR on tumor cells (blue), IgA (black), IgG (pink), plasma cells (red), additional B cells (green), and unstained cells (gray). Each core has a border that matches the cluster it belongs to (see the Cluster Annotations live panel). Representative cores from each of the clusters dominated by pIgR, IgA, and IgG are shown. (B) t-SNE scatterplot showing the marker spread computed from the 210 x 5 count matrix prior to clustering.

A recent study on endometrial cancer^28^ explored the effects of coordinated humoral response (from plasma cells) and cellular immune responses (from T and B cells) in the progression of four different human endometrial cancer subtypes: clear cell carcinoma, serous, endometrioid type high grade, and endometrioid type low grade. These effects were studied by investigating the spatial colocalization and co-expression of polymeric immunoglobulin receptor (pIgR) by tumor cells with immunoglobulins A and G (IgA, IgG) secreted by B cells. The imaging data in this study consisted of 210 tissue microarray (TMA) cores from endometrial tumor samples stained for plasma cells, B cells, IgA, IgG, and pIgR. Each TMA core was available as a 7-marker TIFF file from which we quantify the number of cells coexpressing pIgR (blue), IgA (black), and IgG (pink) over tumor beds and produce a .png file for each core depicting cells with colocalized markers. We use Mistic to visualize these TMA cores arranged in rows (Figure 10). Next, we construct a 210 × 5 count matrix where each row is a core and each column entry in a row consists of the counts for each marker per core. This matrix is clustered using a GMM. In Figure 11A, we see the clusters and corresponding t-SNE scatterplot on the live canvas and the image t-SNE rendered using Mistic. We also show representative cores from each of the three clusters that are high in pIgR, IgA, and IgG. The image t-SNE clearly displays the presence of marker variations across the clusters. For example, it is evident that cores present at the extremities of the t-SNE branches highly express one of pIgR, IgA, or IgG. This was additionally confirmed by plotting the marker distribution spread on the t-SNE scatterplot (Figure 11B). Further, by visual inspection of the image t-SNE, we note that pIgR (blue) was more abundant than IgG (pink) followed by IgA (black). Comparing and visualizing the relevant cores through Mistic in this manner emphasized that the cells colocalizing pIgR and IgA were sparser than cells with pIgR and IgG, and this guided further downstream spatial analysis to take the phenotypic sparsity into account. It was also evident that the four cancer types expressed these marker colocalization patterns similarly (Figures S5A and S5B). These insights derived from Mistic combined with detailed statistical analysis helped reinforce the findings in the study that IgA was a key player in predicting survival and that immune protection, leading to better survival, was associated with tumors with pIgR + IgA and, to a lesser extent, pIgR + IgG.

#### CODEX (PhenoCycler) tumor images

We generate a stack montage of all 32 channels of the CODEX (PhenoCycler) Human FFPE Tonsil data^41^ (Figure S6). We also use 88 duplicate images of the 64-channel CODEX Human FFPE breast adenocarcinoma and test Mistic on seven channels: Keratin14, FoxP3, CD34, CD8, CD3e, CD68, and perlecan (Figure S7). Further we tested Mistic on 105 copies of the unpublished CODEX 16-channel multiplex pyramidal tiff (QPTIFF) tonsil dataset that was provided by Akoya Biosciences (Figure S8). QPTIFF is a Vectra-compatible format.^49^

#### CyCIF images of colorectal carcinoma

We use three CyCIF images from colorectal carcinoma.^42^ The images are in OME-TIFF format,^48^ 7 GB in size with dimensions 1,344 × 1,792, and have four marker channels. Figure S9A shows the Mistic static canvas where 14 duplicates of each of the three CyCIF images (DAPI, CD3, CD4, CD8, CD20, CD68, FoxP3) are rendered randomly, and Figures S9B–S9D are the composite thumbnails generated by Mistic for the three CyCIF images.

In Table 2, we summarize the different datasets, the data formats, and dimensions used in this section to test Mistic for generalizability and scalability.

## Discussion

Understanding the complex ecology of a tumor tissue and the spatiotemporal relationships between its cellular and microenvironment components is becoming a key component of translational research, especially in immuno-oncology. The generation and analysis of multiplexed images from patient samples is of paramount importance to facilitate this understanding. In Table 1, we highlight different image viewers currently available as open-source or commercial software. While most software can handle the visualizing and processing of a single multiplex or microscopy image, to our knowledge, there exists no current image viewer allowing the simultaneous preview of multiple multiplexed images, rendered using t-SNE coordinates or random coordinates. Mistic does not provide additional image processing capabilities such as adjusting images for brightness, sharpness, etc., or detecting objects (segmentation), since Mistic was built with the motivation of providing a preliminary all-image view to aid in better informing quantitative downstream analysis such as identifying spatial patterns across the tumor-immune environment (sections image t-SNE-based visualization of multiplexed images from a NSCLC cohort shows marker expression clustering across different patient response groups and tissue microarray cores for endometrial cancer) and in visualizing specific marker channels (section t-CyCIF image of primary lung squamous cell carcinoma). Using the visuals from Mistic, selected single multiplexed images can be further analyzed using established software in Table 1. Software such as ml4A,^32^ Mirador,^33^ and OpenSeadragon^34^ currently do not cater to 2D multiplexed images. Mistic aims to fill this gap by providing this simple functionality to view multiple images at once, while also giving users the option to view images based on a set of user choices. In our test runs using 92 images (with dimension 1,024 x 1,024 pixels), Mistic takes under a minute to process and render the images according to the user options available (for user options, see section image t-SNE rendered through the static canvas and Figure 2A).

As part of future work, a few potential improvements will be introduced to Mistic. Once a set of images are identified using Mistic, we would like to render those images separately in the live panel. This gives the user an additional perspective to refine the selected images for further analysis. We also hope to integrate Mistic into one of the open-source software viewers listed in Table 1. This would require the development of an additional framework in React JavaScript,^50^ which is the single largest user interface framework.

Through our generalized examples of NSCLC (consisting of 92 7-marker Vectra TIFF images from nine patients), lung adenocarcinoma (70 44-marker t-CyCIF OME-TIFF images), colorectal carcinoma (42 4-marker CyCIF OME-TIFF images), breast adenocarcinoma (88 64-marker CODEX OME-TIFF images), tonsil data (105 16-marker CODEX QPTIFF images), and endometrial cancer (210 PNG images from 107 patients), we have demonstrated the functionality and practicality of Mistic. Our aim is that Mistic will be used as a first step to viewing multiplexed images simultaneously. This all-image visual preview should facilitate preliminary insights into possible marker expression patterns, aiding downstream image analysis for predicting disease progression and identifying clinical biomarkers.

## Experimental procedures

### Resource availability

#### Lead contact

Further information and requests for resources should be directed to and will be fulfilled by the lead contact, Alexander R. A. Anderson (alexander.anderson@moffitt.org).

#### Materials availability

This study did not generate new samples.

#### Data and code availability

**Section 1: Data**
• The t-CyCIF lung data are publicly available.^38^, ^39^, ^40^
• The CODEX (PhenoCycler) data are publicly available.^41^
• The Akoya 16-plex multiplex file format (QPTIFF) tonsil demo dataset for CODEX was provided to us by Akoya Biosciences.
• The NSCLC images reported in this study cannot be deposited in a public repository because they are unpublished and shall be made available upon reasonable user request. To request access, contact lead contact, Alexander R. A. Anderson (alexander.anderson@moffitt.org).
• As an example dataset, we have deposited an anonymized subset of 10 FoVs from the NSCLC dataset here: https://doi.org/10.5281/zenodo.6131933.
• The endometrial cancer images reported in this work can be made available by contacting the lead author of the study.^28^
• The human colorectal cancer is publicly available.^42^

**Section 2: Code**
All original code has been deposited at Zenodo: https://doi.org/10.5281/zenodo.5912169 and is publicly available as of the date of publication.
Mistic is also downloadable at https://github.com/MathOnco/Mistic. Instructions regarding installation, setup, and code deployment can be found at https://mistic-rtd.readthedocs.io. The code is written in Python 3.6 and uses Bokeh, which is a Python library for creating interactive visualizations for modern web browsers. Mistic is indexed on PyPI and requires Python ≥3.6. Code is available under the MIT license. Minimum CPU/memory specifications that Mistic has been tested on are as follows: CPU: Intel Core i9; CPU speed: 2.4 GHz; number of CPUs: 1; total number of cores: 8; RAM: 32 GB.

**Section 3:**
Any additional information required to reanalyze the data reported in this paper is available from the lead contact upon request.

## Supplemental information

Document S1. Figures S1–S9

Document S2. Article plus supplemental information
