# Supplementary material for: Mistic: An open-source multiplexed image t-SNE viewer
Source: Patterns (N Y). 2022 Jun 2;3(7):100523. doi: 10.1016/j.patter.2022.100523 (PMC9278502; doi:10.1016/j.patter.2022.100523)
Supplement: Document S1. Figures S1–S9 [file mmc1.pdf]

Patterns, Volume *3*

Supplemental information

Mistic: An open-source multiplexed image t-SNE viewer

Sandhya Prabhakaran, Chandler Gatenbee, Mark Robertson-Tessi, Jeffrey West, Amer A. Beg, Jhanelle Gray, Scott Antonia, Robert A. Gatenby, and Alexander R.A. Anderson

Supplemental figures

Figure legends

Figure S1. **The static and live canvases of Mistic showing image metadata**. The static and live canvases of Mistic showing image borders colored based on additional metadata provided by the user such as treatment (A), cluster annotations (B) or patient ids (C) for the NSCLC example.

Figure S2. Mistic tested on the 44-channel Lung adenocarcinoma lymph t-CyCIF data[1-3] for 50 image repeats. The static canvas shows 50 repeats of the Lung t-CyCIF image arranged in rows. Mistic gives the composite image using 6 markers (CD45, Keratin, α-SMA, FoxP3, PD-1, PD-L1). The live canvas shows a hover toolbox with additional information for an image.

Figure S3. **Mistic tested on the 44-channel Lung adenocarcinoma lymph t-CyCIF data[1-3] for 60 image repeats.** The static canvas shows 60 repeats of the Lung t-CyCIF image based on random co-ordinates. Mistic gives the composite image using 6 markers (CD45, Keratin, α-SMA, FoxP3, PD-1, PD-L1). The live canvas shows a hover toolbox with additional information for an image.

Figure S4. **Mistic tested on the 44-channel Lung adenocarcinoma lymph t-CyCIF data[1-3] for 70 image repeats.** The static canvas shows 70 repeats of the Lung t-CyCIF image arranged in rows. Mistic gives the composite image using 6 markers (CD45, Keratin, α-SMA, FoxP3, PD-1, PD-L1). The live canvas shows proxy cluster assignments.

Figure S5. **210 Endometrial Tumor microarray (TMA) cores[4] stained for pIgR on tumor cells, IgA, IgG, plasma cells, and additional B-cells**. **A**. t-SNE layout for these 210 cores showing the spread of the four endometrial cancer subtypes: clear cell carcinoma (CCC), serous (S), endometrioid type high grade (EH), and endometrioid type low grade (EL), with color legend. Each t-SNE dot represents a core. **B**. Corresponding image t-SNE layout of the 210 cores showing the distribution of the four endometrial cancer subtypes with image border colors corresponding to the subtypes.

Figure S6. **Stack Montage rendered by Mistic for the Human FFPE Tonsil CODEX (PhenoCycler) data[5] with 32 markers. A.** The static canvas shows all 32 markers and the live canvas shows the t-SNE scatter plot. We identify the CD21 channel using the live canvas (shown with hover tool details) and highlight the CD21 thumbnail in yellow in the static canvas. **B.** The zoomed in CD21 thumbnail (file name is obtained from the hover tool) and **C.** The CODEX CD21 channel as viewed using FIJI[6]. FIJI provides the single marker views for 32 markers whereas with Mistic we can view all 32 markers as a montage.

Figure S7. **Mistic tested on the 64-channel Human FFPE Breast adenocarcinoma CODEX (PhenoCycler) data[5]. A.** The static canvas shows 88 repeats of the CODEX image arranged in proxy user-generated t-SNE co-ordinates. Mistic gives the composite image using 7 markers (Keratin14, FoxP3, CD34, CD8, CD3e, CD68 and Perlecan). **B.** The zoomed in composite thumbnail generated by Mistic and **C.** The CODEX image[7] for Keratin19, CD8, CD3e, Vimentin, CD68 and CollagenIV. Note that with Mistic we can view any number of markers at once.

Figure S8. **Mistic tested on the 16-channel Human FFPE Tonsil CODEX (PhenoCycler) pyramidal TIFF (QPTIFF) data[8] provided by Akoya Biosciences.** Note that we read in all the five image pyramids in the file and use the fourth image pyramid for rendering in Mistic. **A.** The static canvas shows 105 repeats of the CODEX image arranged in proxy user-generated t-SNE co-ordinates. Mistic gives the composite image using 6 markers (PanCK, CD31, SMA, Ki67, CD8 and CD20). **B.** The zoomed in composite thumbnail generated by Mistic and **C.** The CODEX image for PanCK, CD31, Ki67 and CD20 rendered using QuPath[9]. Note that with Mistic we can view any number of markers at once.

Figure S9. **Mistic tested on 4-channel Human colorectal carcinoma CyCIF (OME-TIFF) images[10]. A.** The static canvas shows 42 duplicates of the three CyCIF images (DAPI, CD3, CD4, CD8, CD20, CD68,

FoxP3) rendered randomly. **B-D.** The zoomed-in composite thumbnails generated by Mistic for the three CyCIF images.

Figures

Figure S1

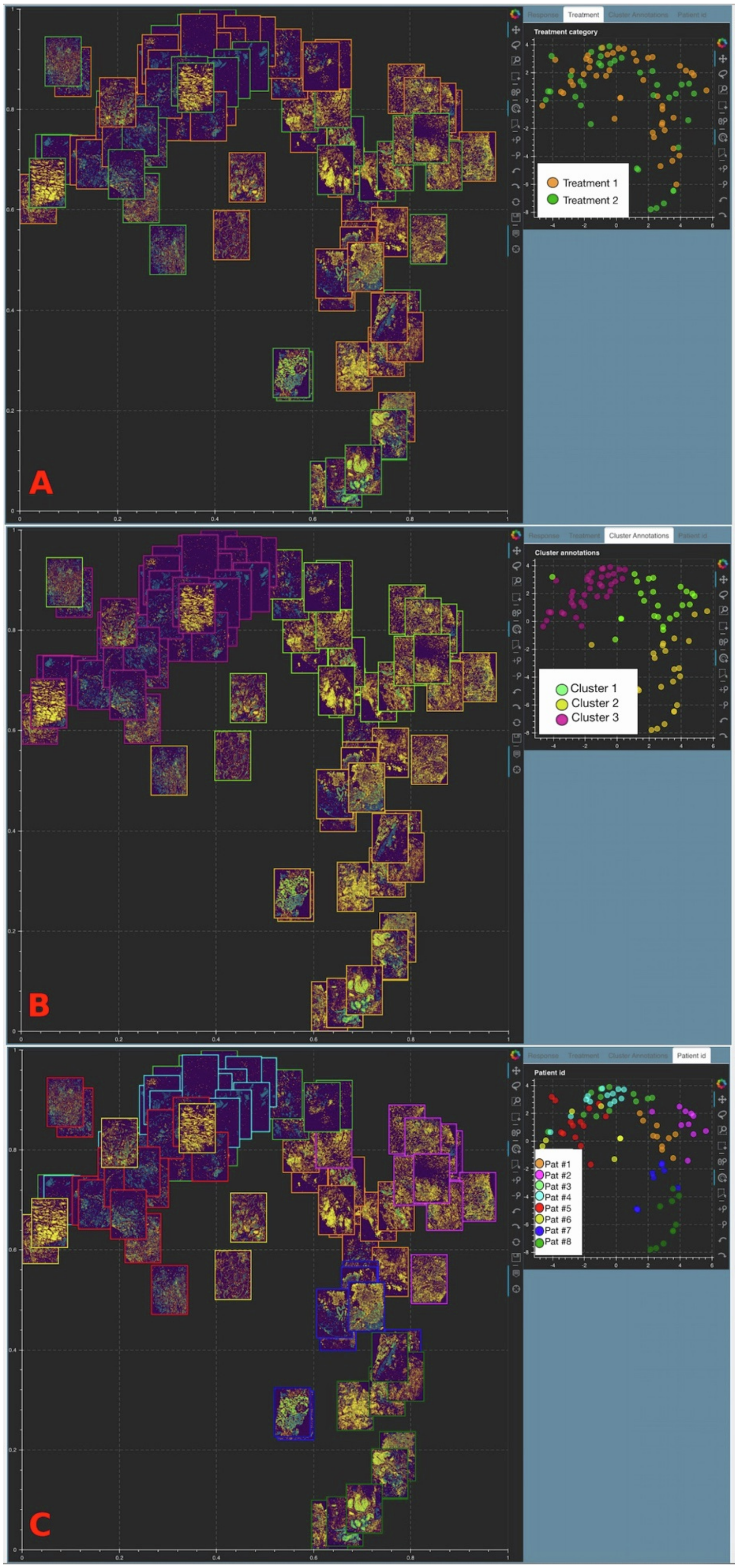
A
Treatment
Treatment category
Treatment 1
Treatment 2
B
Cluster Annotations
Cluster annotations
Cluster 1
Cluster 2
Cluster 3
C
Patient id
Pat #1
Pat #2
Pat #3
Pat #4
Pat #5
Pat #6
Pat #7
Pat #8

Figure S2

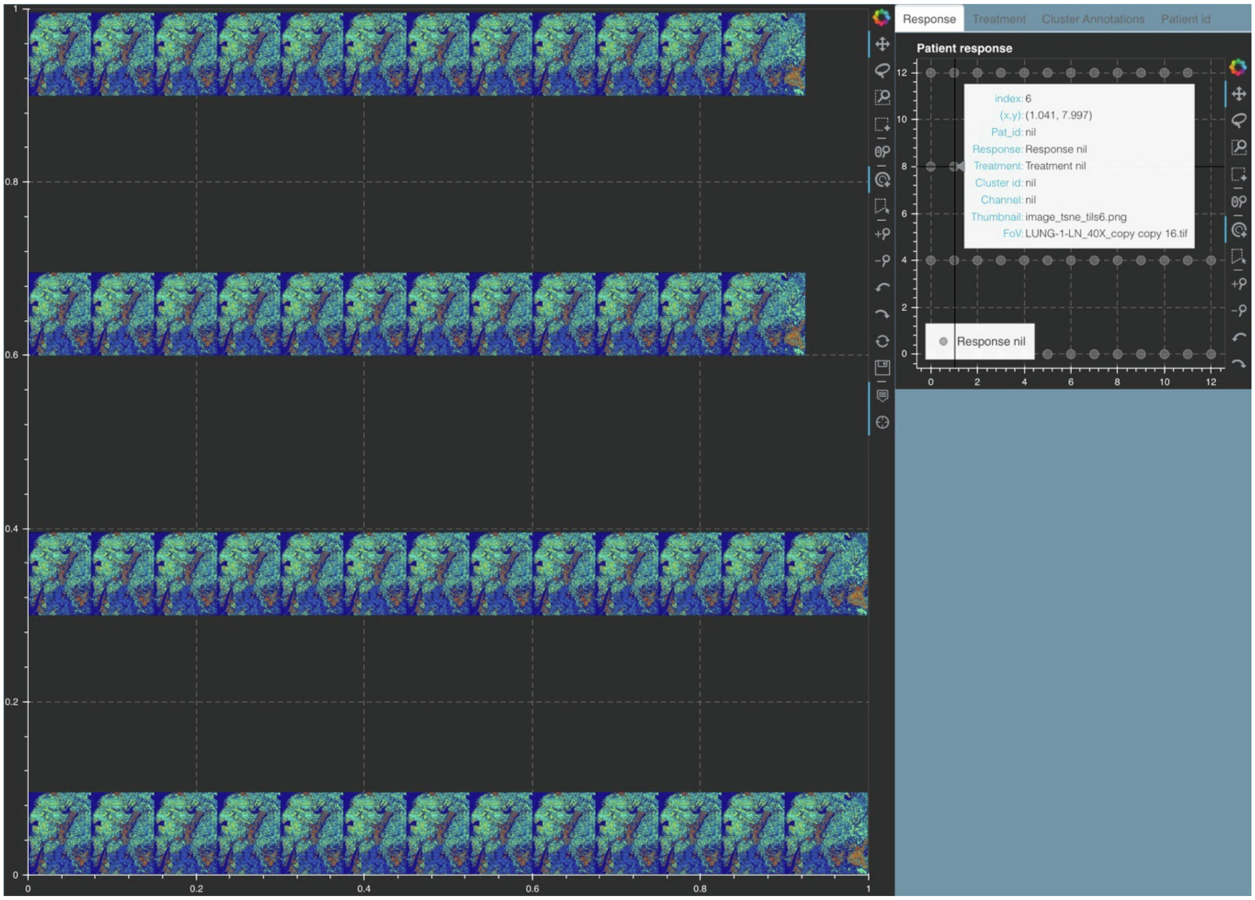

Response
Treatment
Cluster Annotations
Patient id
Patient response
index: 6
(x,y): (1.041, 7.997)
Pat_id: nil
Response: Response nil
Treatment: Treatment nil
Cluster id: nil
Channel: nil
Thumbnail: image_tsne_tils6.png
FoV: LUNG-1-LN_40X_copy copy 16.tif
Response nil

Figure S3

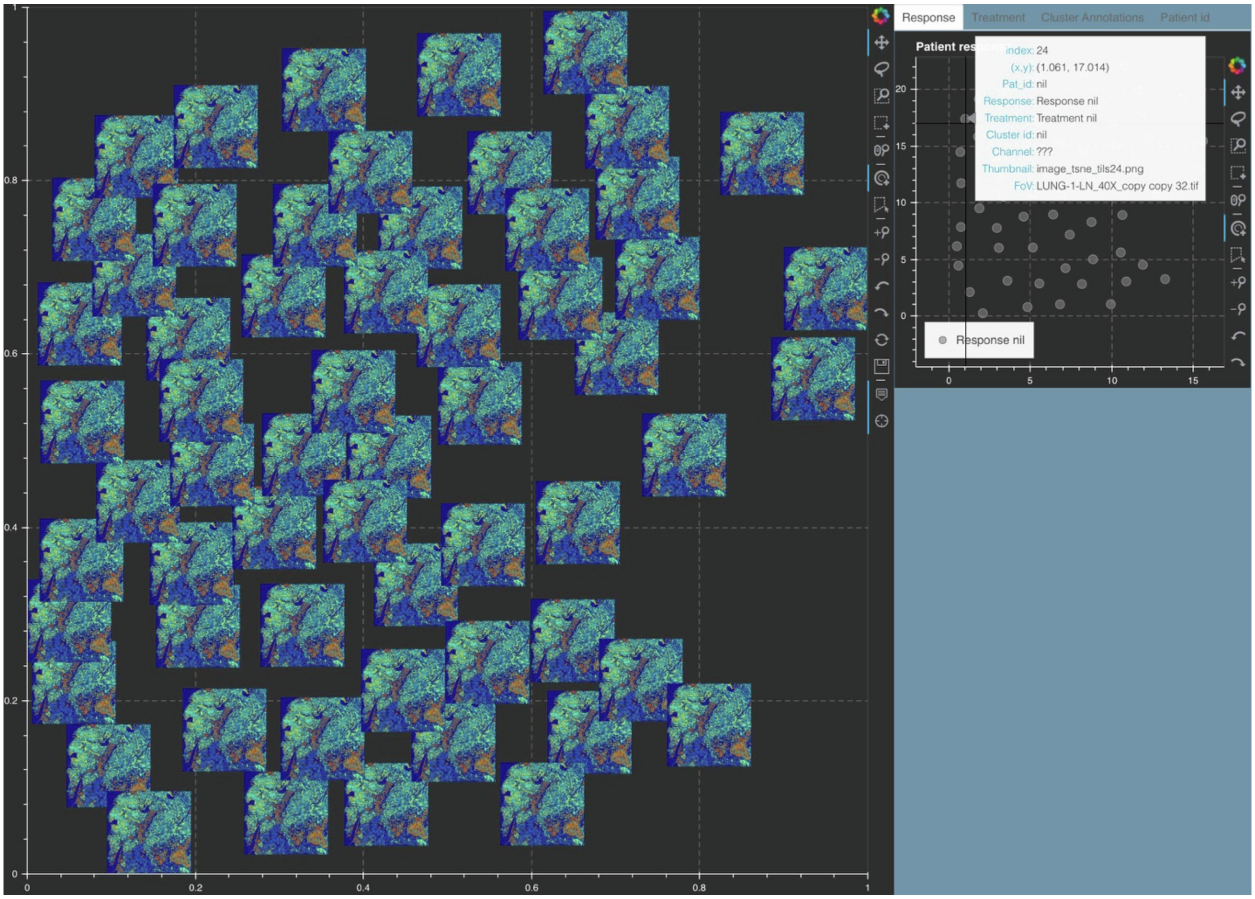

Response
Treatment
Cluster Annotations
Patient id
Patient res
index: 24
(x,y): (1.061, 17.014)
Pat_id: nil
Response: Response nil
Treatment: Treatment nil
Cluster id: nil
Channel: ???
Thumbnail: image_tsne_tils24.png
FoV: LUNG-1-LN_40X_copy copy 32.tif
Response nil
0
5
10
15
20
0
0.2
0.4
0.6
0.8
1

Figure S4

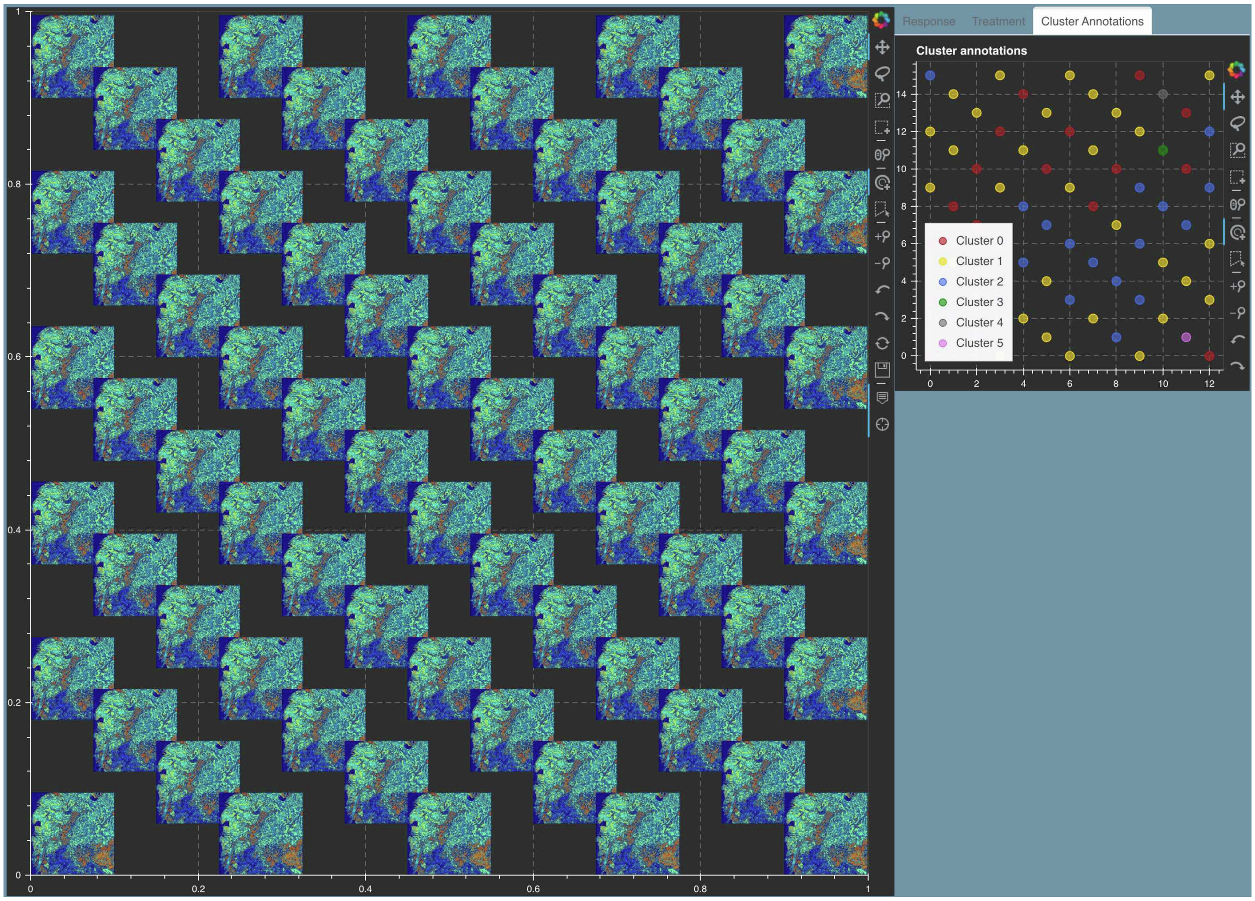
Response
Treatment
Cluster Annotations
Cluster annotations
Cluster 0
Cluster 1
Cluster 2
Cluster 3
Cluster 4
Cluster 5
0
0.2
0.4
0.6
0.8
1
0
2
4
6
8
10
12
14

Figure S5

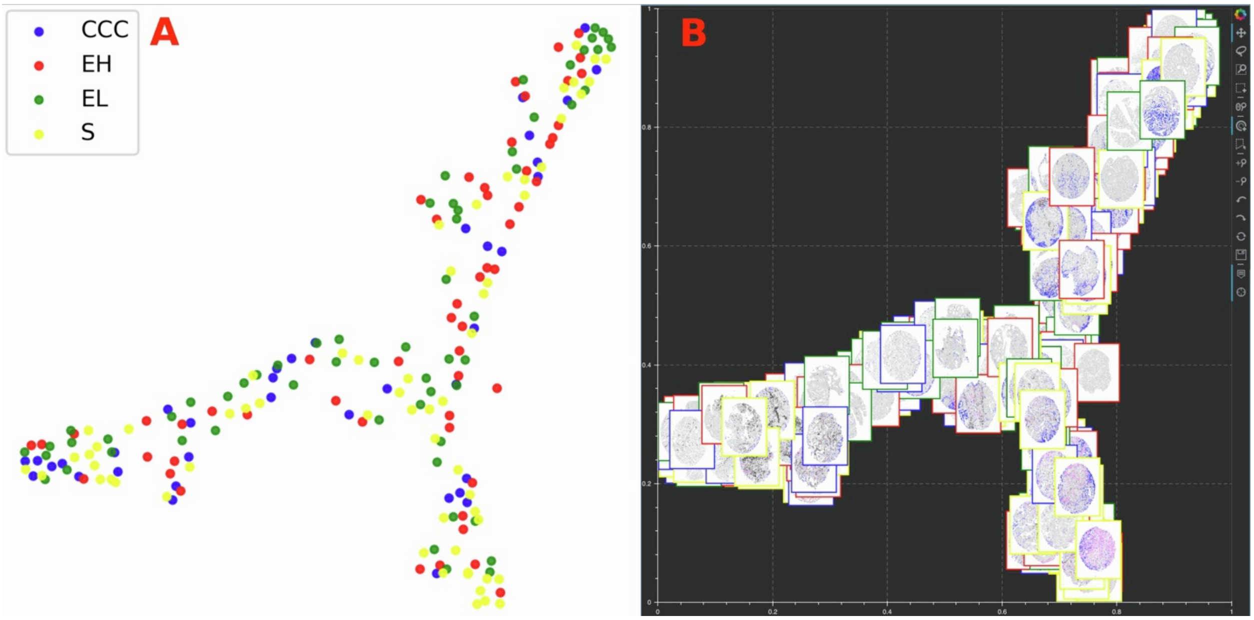

Figure S6

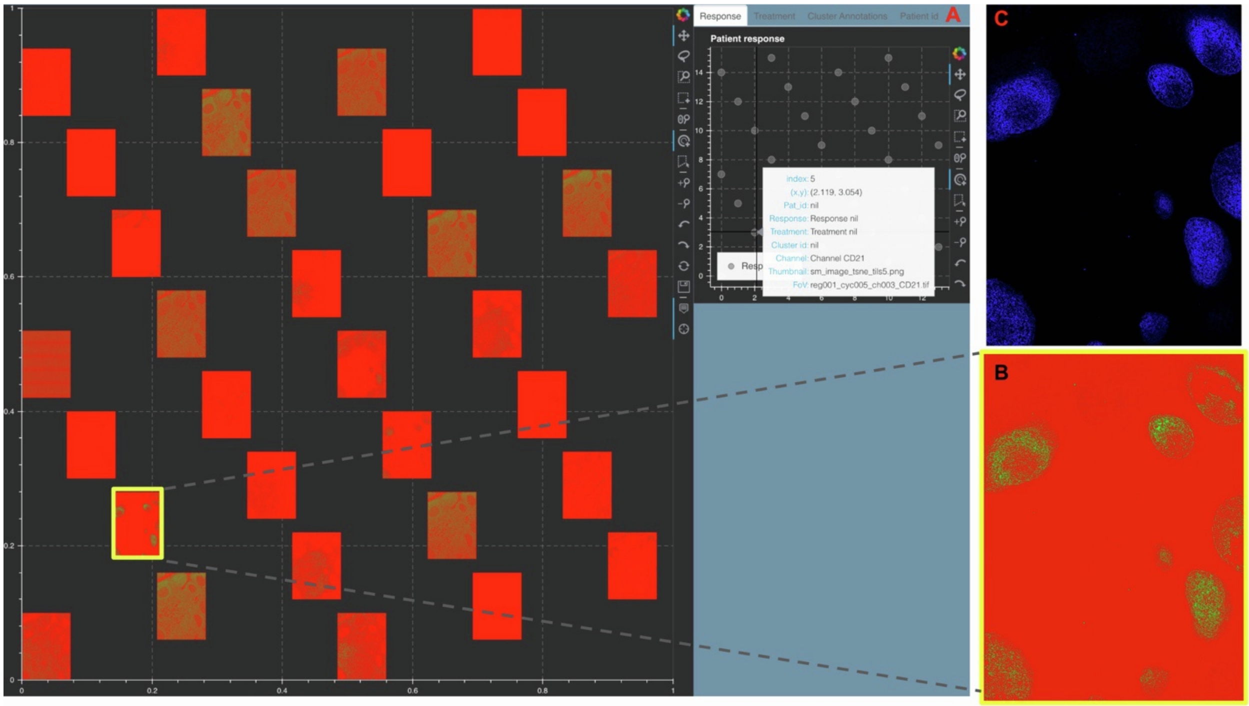
A
B
C
Response
Treatment
Cluster Annotations
Patient id
Patient response
index: 5
(x,y): (2.119, 3.054)
Pat_id: nil
Response: Response nil
Treatment: Treatment nil
Cluster id: nil
Channel: Channel CD21
Thumbnail: sm_image_tsne_tils5.png
FoV: reg001_cyc005_ch003_CD21.tif

Figure S7

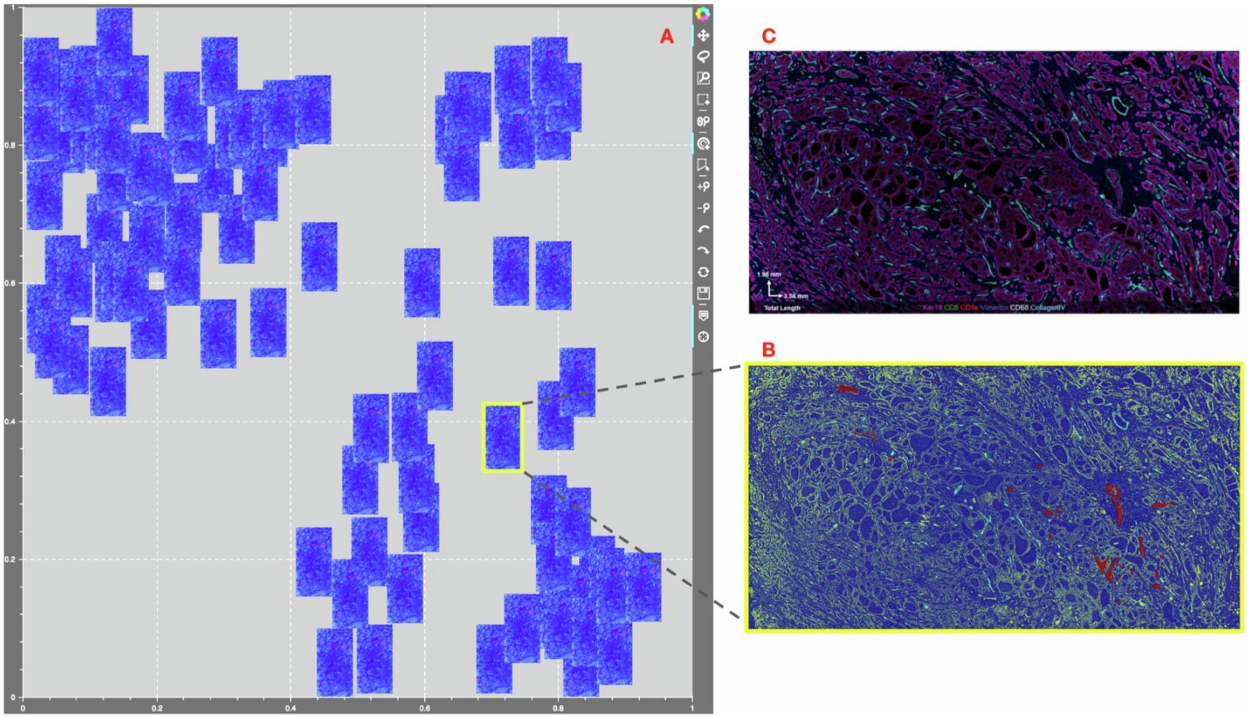
A
B
C
1.90 mm
3.56 mm
Total Length
Ker19 CD8 CD3e Vimentin CD68 CollagenIV
0
0.2
0.4
0.6
0.8
1

Figure S8

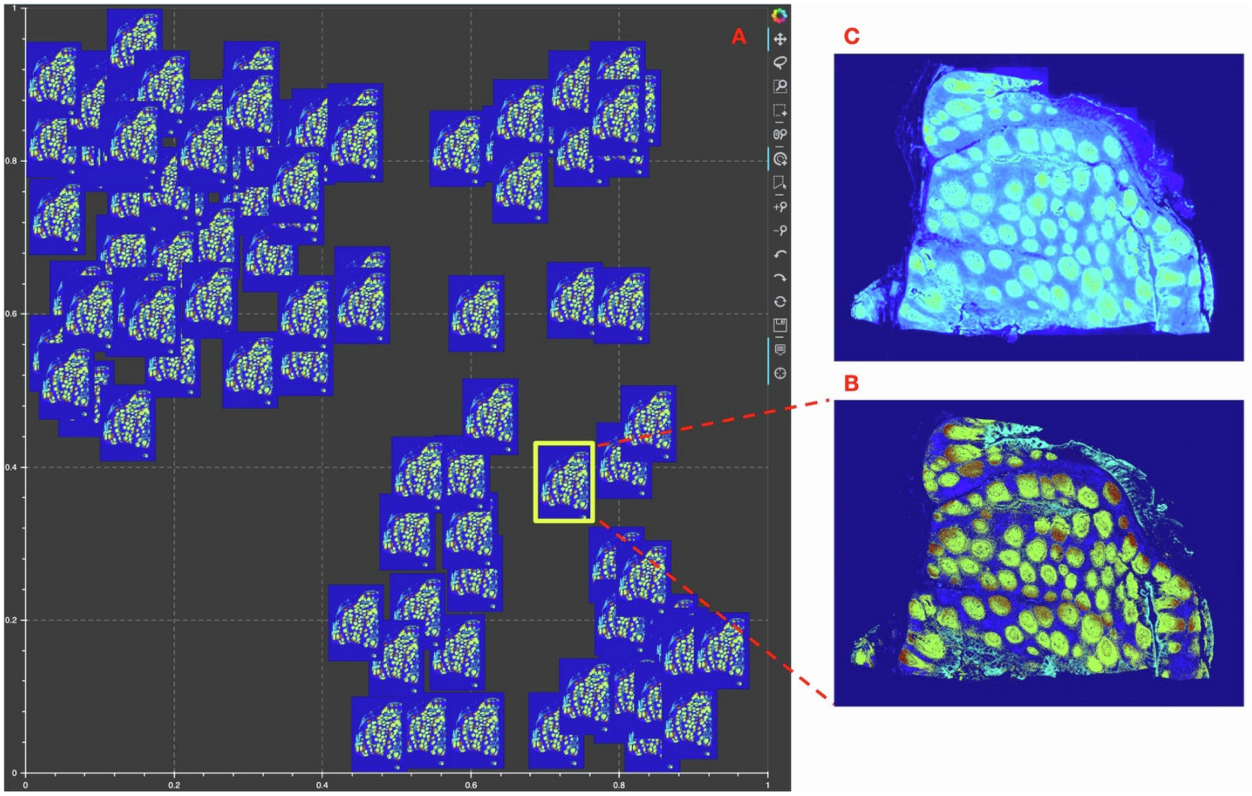

Figure S9

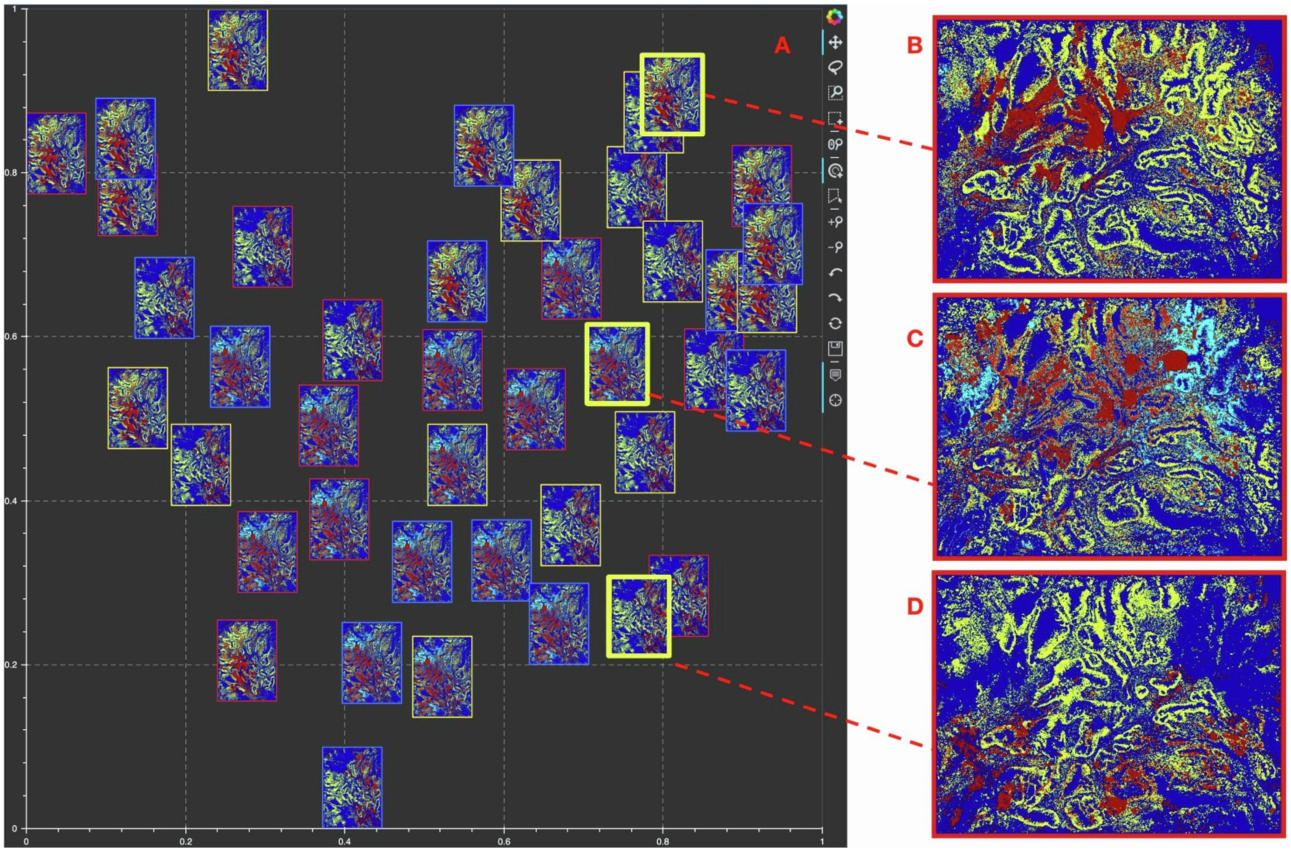

References

[1] Rashid, R., Gaglia, G., Chen, Y.A., Lin, J.R., Du, Z., Maliga, Z., Schapiro, D., Yapp, C., Muhlich, J., Sokolov, A. and Sorger, P., (2019). Highly multiplexed immunofluorescence images and single-cell data of immune markers in tonsil and lung cancer. Scientific Data, 6(1), pp.1-10. https://doi.org/10.1038/s41597-019-0332-y

[2] t-CyCIF data, “https://www.synapse.org/#!Synapse:syn17865732/wiki/592782,” [Online].

[3] t-CyCIF images, “https://www.cycif.org/data/du-lin-rashid-nat-protoc-2019/,” [Online].

[4] Mandal, G., Biswas, S., Anadon, C. M., Yu, X., Gatenbee, C. D., Prabhakaran, S., Payne, K. K., Chaurio, R. A., Martin, A., Innamarato, P., Moran, C., Powers, J. J., Harro, C. M., Mine, J. A., Sprenger, K. B., Rigolizzo, K. E., Wang, X., Curiel, T. J., Rodriguez, P. C., Anderson, A., Saglam, Ozlen., and Conejo-Garcia, J. R. (2022). IgA-dominated humoral immune responses govern patients' outcome in endometrial cancer. Cancer Research, 82(5), 859–871. https://doi.org/10.1158/0008-5472.CAN-21-2376

[5] CODEX data, “https://help.codex.bio/codex/mav/public-datasets,” [Online].

[6] Schindelin, J., Arganda-Carreras, I., Frise, E., Kaynig, V., Longair, M., Pietzsch, T., Preibisch, S., Rueden, C., Saalfeld, S., Schmid, B., Tinevez, J. Y., White, D. J., Hartenstein, V., Eliceiri, K., Tomancak, P., & Cardona, A. (2012). Fiji: an open-source platform for biological-image analysis. Nature Methods, 9(7), 676–682. https://doi.org/10.1038/nmeth.2019

[7] Akoya Biosciences, “https://www.akoyabio.com/blog/36-plex-codex-breast-cancer-ffpe-panel-recapitulates-published-findings/#1593630922137-4a0df0aa-c16c,” [Online].

[8] PerkinElmer, “https://docs.openmicroscopy.org/bio-formats/5.9.2/formats/perkinelmer-vectra-qptiff.html,” [Online].

[9] Bankhead, P., Loughrey, M. B., Fernández, J. A., Dombrowski, Y., McArt, D. G., Dunne, P. D., McQuaid, S., Gray, R. T., Murray, L. J., Coleman, H. G., James, J. A., Salto-Tellez, M., & Hamilton, P. W. (2017). QuPath: Open source software for digital pathology image analysis. Scientific Reports, 7(1), 16878. https://doi.org/10.1038/s41598-017-17204-5

[10] CyCIF data, "https://github.com/MathOnco/valis/tree/main/examples/example_datasets/cycif," [Online].
